# Machine learning models incorporating somatic and mental comorbidities for prolonged length-of-stay prediction in a maximum care university hospital

**DOI:** 10.1186/s12911-025-03290-3

**Published:** 2025-11-26

**Authors:** Sophia Stahl-Toyota, Ivo Dönnhoff, Ede Nagy, Achim Hochlehnert, Inga Unger, Julia Szendrödi, Norbert Frey, Patrick Michl, Carsten Müller-Tidow, Dirk Jäger, Hans-Christoph Friederich, Christoph Nikendei

**Affiliations:** 1 Department of General Internal Medicine and Psychosomatics, Medical University Hospital, Heidelberg, Germany; 2 Department of Medicine Controlling, Medical University Hospital, Heidelberg, Germany; 3 Department of Internal Medicine , Nursing Management, Medical University Hospital, Heidelberg, Germany; 4 Department of Endocrinology and Clinical Chemistry, Medical University Hospital, Heidelberg, Germany; 5 Department of Cardiology, Angiology and Pneumology, Medical University Hospital, Heidelberg, Germany; 6 Department of Gastroenterology, Hepatology and Infectious Diseases, Medical University Hospital, Heidelberg, Germany; 7 Department of Hematology, Oncology and Rheumatology, Medical University Hospital, Heidelberg, Germany; 8 Department of Medical Oncology, Medical University Hospital, National Center for Tumor Diseases, Heidelberg, Germany

**Keywords:** Health care economics, Public health, Psychiatric disorders, Mental health, Multimorbidity, Somatic comorbidity, Internal medicine, Machine learning

## Abstract

**Background:**

Knowledge about the influencing factors on hospital in-patient length-of-stay is integral for optimizing care and resource planning. Many existing studies on prolonged length-of-stay prediction choose a single threshold for the number of days that classifies the length-of-stay as prolonged. The analyses are based on either very heterogeneous or specific cohorts. Most studies take somatic comorbidities into account, while only a few incorporate mental comorbidities.

**Objectives:**

(I) After which timeframe does the number of days of inpatient treatment indicate a prolonged length-of-stay if the threshold for outliers is computed department-wise in a maximum care internal medicine university hospital? (II) How accurately can machine learning models predict prolonged length-of-stay in internal medicine patients? (III) Which mental and somatic comorbidities have the strongest influence on length-of-stay prediction?

**Methods:**

*N* = 28,536 internal medicine cases treated as inpatients at the German University Hospital in Heidelberg in the years 2017 to 2019 comprised the study population. For each of six internal medicine departments, the threshold for prolonged length-of-stay was computed based on median absolute deviation. Department-wise machine learning models for prolonged length-of-stay classification (Random Forest, XGBoost, LightGBM, Logistic Regression) were built on 80% train data employing cross-validation and the Optuna framework for hyperparameter optimization. Model performance was assessed on 20% test data mainly by Area under the Receiver Operator Curve (AUROC). The models incorporated features derived from demographics and mental as well as somatic comorbidities.

**Results:**

Length-of-stay was classified as prolonged if the number of days at the hospital equaled or exceeded 9 (Cardiology), 10 (General and Psychosomatics, Gastroenterology, Medical Oncology), 11 (Endocrinology) or 26 (Hematology). With AUROC = 0.89 the random forest for the Department of Hematology had the highest predictive power, the random forest for the Department of General and Psychosomatic with AUROC = 0.72 the lowest. The variables with strongest influence on prediction comprised the number of somatic comorbidities, the age at diagnosis, mental and somatic comorbidity subgroups. Among the mental comorbidities, stress-related adjustment disorder was the most prominent factor.

**Conclusions:**

Consideration of department-level factors is recommended for prolonged length-of-stay prediction models. Mental as well as somatic comorbidities were among the most relevant factors for the prediction of prolonged length-of-stay and require adequate treatment and reimbursement opportunities.

**Supplementary Information:**

The online version contains supplementary material available at 10.1186/s12911-025-03290-3.

## Background

Ensuring quality of care at hospitals is in the individual interest of the patients seeking treatment as well as the collective interest of the health economic systems covering associated costs [1, 2]. Quality of care at a hospital can be measured across different dimensions. Among them are hospital length-of-stay, rehospitalization rates, non-home discharge and in-hospital mortality [3–5].

Length-of-stay refers to the number of days that an in-patient stayed at the hospital from admission to release during a single administrative case [1]. If a patient’s stay lasts longer than expected, it may be an indication that patient well-being has not improved as quickly as hoped for, and that the hospital`s costs exceeded those costs anticipated for the health needs in this particular case. Identifying the reasons for extended length-of-stay is therefore of great interest from a medical as well as an economic perspective and is the focus of this study.

A unified framework for presenting research on length-of-stay prediction was proposed by Stone et al. [1]. The approaches for length-of-stay analysis include arithmetic methods and data mining methods. In a previous study [6], we reported the application of arithmetic methods, such as average and median length-of-stay and regression analysis for the identical cohort examined in the present study. To further explore the specific factors that lead to longer length-of-stay, applying data mining methods by treating the problem as a classification into prolonged or not prolonged length-of-stay is promising and has been modeled in a similar manner by many studies.

Table 1 depicts a collection of studies that predicted prolonged length-of-stay. This collection was identified by weekly manual review of all articles that matched a literature alert set in PubMed for the search ‘length-of-stay[Title/Abstract] OR “length of stay“[Title/Abstract]’ in the year 2024 as well as selected references mentioned in recent reviews [1, 2, 7]. Analyzing length-of-stay as a classification problem into the classes prolonged versus not prolonged raises the question of how exactly the anticipated length-of-stay is to be determined. The threshold chosen in the literature for the number of days at a hospital for a case to be counted as prolonged ranged from ≥ 2 to ≥ 25 days. This collection includes only studies for general departments, not emergency departments, for which length-of-stay studies refer to the range of hours. The reasons stated for the choice of prolonged length-of-stay thresholds included mostly measures pertaining to the cohort or index disease, such as average or median length-of-stay, or the application of more complex formulas such as Tukey’s criterion for outlier definition [8]. Several studies mentioned other references or did not report a justifying explanation. The decision seemed to depend on the research question, however, clinical reasoning for the choice of the threshold was rarely provided.

**Table 1:** Studies predicting prolonged length-of-stay with explanations for thresholds. Sorted ascending by PLOS threshold. PLOS: prolonged length-of-stay. AUROC: area under the receiver operator curve. ACC: Accuracy. SHAP: SHapley additive exPlanations. LIME: local interpretable Model-agnostic Explanations. RNN: recurrent neural network

Citation	Country of sample	*N* samples	Years sample collected	Inclusion criteria	PLOS threshold [days]	PLOS threshold explanation	AUROC max for PLOS	Feature Importance Method
Chirongoma 2024 [16]	USA	1048	2008 to 2018	Procedure total ankle arthroplasty	≥ 2	average LOS for this procedure	0.748	Permutation feature importance
Bopche 2024 [17]	Norway	35,591	2015 to 2020	Suspected bloodstream infection	≥ 3	No reason stated	0.9797	SHAP
Shalaby 2024 [18]	Saudi Arabia	3081	2015 to 2020	Female, acute myocardial infarction	≥ 5	average LOS for this sample	NA	NA
Berg 2024 [19]		816	2016 to 2018	Primary Fusion for Congenital Scoliosis	≥ 5	median LOS for this sample	NA	NA
Jain 2024 [20]	USA (SPARCS)	2,306,668	2016	All inpatients at New York State Hospitals	≥ 5	Last of 5 balanced classes	0.964	SHAP
Webber 2024 [4]	USA	1447	2013 to 2019	Prodedure major colorectal operation	≥ 7	No reason stated	NA	NA
Karabacak 2024 [21]	USA	6999	2014 to 2020	Intracranial meningioma	≥ 7	75% percentile LOS for this sample	0.845	SHAP
Wang 2023 [22]	China	677	2017 to 2019	Acute ischemic stroke	≥ 7	No reason stated	0.857	SHAP
Chrusciel 2021 [11]	France	5006	2019	Admitted through emergency department	≥ 7	median LOS for this sample	Not reported, ACC = 0.76	Not reported
Zhang 2020 [23]	USA (MIMIC-II)	39,429	2001 to 2012	All adult admissions longer than 24 h	≥ 7	Two references	0.784	Not reported
Rajkomar 2018 [5]	USA	216,221	2009 to 2016	All adult admissions longer than 24 h	≥ 7	75th percentile LOS for this sample	0.86	Attention mechanism, RNN weighting
Santos 2024 [24]	Portugal	66,661	2008 to 2015	Chronic Obstructive Pulmonary Disease	≥ 8	median LOS for this sample	NA	NA
Han 2021 [25]	China	1298	2016 to 2019	Procedure total knee arthroplasty	≥ 8	median LOS for this sample	0.766	Classifier evaluator
Wegiel 2018 [26]	Poland	212	2016 to 2017	Myocardial infarction	≥ 8	median LOS for this sample	NA	NA
Yasin 2024 [27]	China	580	2016 to 2022	Tuberculous spondylitis	≥ 10	75th percentile LOS for this sample	0.86	SHAP, permutation, LIME, Gini
Gilbert 2023 [28]	France	1,042,234	2017	Geriatric, acute care as emergency	≥ 11	As in HFRS study in UK	NA	NA
Jaotombo 2022 [8]	France	73,182	2015	Acute hospitalization	≥ 14	90th percentile for this sample, Tukey’s formula	0.81	Permutation feature importance
Chen 2023 [29]	China	18,195	2017 to 2018	Ischemic stroke	≥ 7, ≥ 14	7-14d protective factor for adverse outcome, insurance policy	Not reported, ACC = 0.89	information gain method
Lai 2024 [30]	Hong Kong	7778	2010 to 2024	Geriatric, fragility fracture	≥ 20	recovery process long	0.84	SHAP
Jaotombo 2023 [15]	USA (MIMIC-II)	30,764	2001 to 2012	All adult admissions longer than 24 h	≥ 25	Tukey’s formula	0.944	Permutation feature importance, LIME
Mittal 2024 [31]	USA (ACS-NSQIP)	23,656	2013 to 2020	Revision total knee arthroplasty	Not reported	75th percentile LOS for this sample	0.75	Not reported

Reasoning for the important choice of the prolonged length-of-stay threshold can be approached from different angles. First, an administrative reason for a 7-day threshold could be related to clinical routine schedules that follow a weekly rhythm, as schedules permitting continuity for hospital staff to be acquainted with patients have shown better outcomes [9]. A second reasoning approach for a 7- or 8-day threshold could involve national or international comparability, as 7.24 days was the overall average length-of-stay across 25 European countries in 2019 [10], and for the 5 countries that have a similar healthcare system as Germany, which was termed Social Health Insurance, the average was 8.31 days. A third approach may aim for balanced classes, e.g. by choosing the median of the cohort [11]. The fourth approach may involve a cohort-related statistic that aims at identifying causes for outliers. Measuring the true length-of-stay that captures different conditions requiring different lengths of diagnostic and treatment regimens, and deriving a statistic to identify the extremes of the distribution reflects the aim of providing adequate care, while identifying risk factors for extremely long staying cases, or outliers. One measure that has been described as especially robust to guard against extreme outliers is the median absolute deviation (MAD) [12–14]. If the threshold is supposed to reflect outliers, a base cohort is needed on which the statistical measure is computed. The existing studies, however, cover only the two extremes of sample populations that were either very large and heterogeneous [8, 15] or incorporated very specific diagnoses or procedures [4].

Besides the specific target definition and the cohort details, the predictors reported in the literature for prolonged length-of-stay classification also showed great variability. They included various combinations and detail levels of socio-demographics, somatic comorbidities, mental comorbidities and other variables. The data sources ranged from laboratory test results and procedural codes to free text discharge summaries.

In the following, we describe those studies in more detail that included somatic as well as mental morbidities in some form as predictors for prolonged length-of-stay to exemplify the heterogeneous landscape of modeling mental diagnostic information and the relationship to length-of-stay that has been described so far. Most of these refer to very specific cohorts. For patients admitted mainly for chronic obstructive pulmonary disease, the presence of any comorbid psychiatric disorder made them more likely to stay longer at the hospital [24]. Division by specific psychiatric disorders revealed that developmental disorders increased adjusted odds ratio for length-of-stay ≥ 8 days the most, followed by mood disorders and neurotic, personality and other nonpsychotic disorders. For patients suspected of bloodstream infections, Bopche et al. incorporated mental comorbidity as number of diagnostic codes in the entire ICD-10 (International Statistical Classification Of Diseases And Related Health Problems, 10th revision) F-chapter for mental disorders and behavioral disorders (ICD_F) in the current or most recent episode as well as a separate feature for cumulative count in complete medical history [17]. In their risk assessment, ICD_F was among the top 10 predictors for prolonged length-of-stay (≥ 3 days). For patients with colorectal surgery, patients with history of psychiatric diagnosis had a higher proportion of hospital stays ≥ 7 days [4]. Even though they presented the demographics of psychiatric subgroups and described extensive efforts in manual review for possibly missed diagnoses and prescribed medications, ensuring the psychiatric diagnosis accuracy, they do not seem to have used those details in the statistical analysis, which based solely on presence or absence of history of psychiatric diagnoses. For prolonged length-of-stay (≥ 20 days) of geriatric fragility fracture patients, the list of important features of the strongest model included mental state as measured by the Montreal Cognitive Assessment 5-min protocol [30]. A very large American study had disease code level information available, but did not report diagnosis details in the feature importances [20].

Considering the above description of literature on prolonged length-of-stay prediction, we see several so far unexplored research opportunities that lead to the objectives of this study. (I) Regarding the threshold for the number of days for which a hospital stay counts as prolonged, previous studies have not described a consistent way of determining the threshold [8]. This question of choosing an adequate threshold is therefore addressed by the first objective of our study, in which we explore the effect of applying a statistical outlier approach on a department specific level. By building prediction models for prolonged length-of-stay by department, the underlying populations were not as heterogeneous as those studies that incorporated an entire hospital into one study population and at the same time not as specific as those studies that focused on a single diagnosis or procedure. (II) The second objective was to determine the discriminatory power of predictive models for prolonged length-of-stay, using administrative diagnosis data that is modeled in a clearly reproducible manner. The influence of mental comorbidity on length-of-stay was previously reported in literature, but rarely on a detailed diagnosis level. Thus, (III) as a third objective, the ranking of detailed mental and somatic comorbidities in their importance for the best performing models were to be compared.

## Methods

### Cohort

*Sample.* We retrospectively examined all inpatient cases that were treated at the Center of Internal Medicine of the University Hospital in Heidelberg, Germany, during the years 2017, 2018 and 2019 [6]. The six departments that were part of this study were the departments of (1) Endocrinology, Diabetology, Metabolism and Clinical Chemistry (Endocrinology), (2) General Internal Medicine and Psychosomatics (General and Psychosomatics), (3) Cardiology, Angiology and Pneumology (Cardiology), (4) Gastroenterology, Hepatology and Infectious Diseases (Gastroenterology), (5) Hematology, Oncology and Rheumatology (Hematology) and (6) Medical Oncology, one core area of the National Center for Tumor Diseases (Medical Oncology). The data source was an export of the electronic medical records of the administrative department, in which diagnostic ICD-10 (International Statistical Classification Of Diseases And Related Health Problems, 10th revision) German Modification codes that were relevant to the respective hospital stay were available. Cases that were medically associated transfers, were direct follow-up admissions, or had overlapping timespans were merged during data preprocessing. These steps yielded *N* = 28,536 cases, defined as one insurance-claim related unit, on the basis of 20,193 patients, defined as one unique person.

*Inclusion Criteria.* Cases were included if the patients (1) were at least 18 years old, (2) had no main diagnosis of the ICD-10 for psychiatric diseases, (3) and had a length-of-stay of at least two days. These inclusion criteria were chosen to focus only on inpatients who stayed overnight and were admitted based on a primarily somatic main diagnosis.

### Variables

*Target variable: prolonged length-of-stay.* To treat length-of-stay as a classification task, a threshold for the number of days for which a case counted as prolonged was needed. Due to inherent differences in treatment approaches and patients admitted across the six departments of internal medicine, it could not be assumed that the length-of-stay would be equal. Therefore, the threshold for prolonged length-of-stay was computed separately within each department as the Median Absolute Deviation (*MAD*) using the formula:

1 $$\:MAD=b*median\:\left(\left|X-median\left(X\right)\right|\right)$$

2 $$\:{threshold}_{plos}=median\left(X\right)+MAD$$

where *X* are the training data observations (see below for data splitting description) and *b = 1.4826* is a constant [12–14]. As opposed to average-based deviation measures, the MAD is known for its robustness in the case of extreme outliers [12, 32].

*Feature variables: Demographic and main diagnosis.* The patients’ gender, age at admission and main diagnosis ICD-10 chapter were considered as potential confounders in all analyses.

*Feature variables: Mental comorbidity.* Mental comorbidity was defined as any secondary ICD-10 diagnosis code from Chapter V (F0-F9) [33]. To explore the detailed effects on the classification tasks, the mental comorbidities were divided into the following subgroups: group F0 was split into “F05 Organic delirium” and “F0x Organic dementia and others” to separate delirium from other organic mental disorders [34]; group F1 was split into “F10 Substance Alcohol”, “F17 Substance Tobacco”, and “F1x Substance others” to differentiate legal from illegal substances; group F3 was split into “F32 Affective Depressive episode” and “F3x Affective recurrent and others” to account for differences in severity represented by a one-time episode in comparison to recurring mood disorders; F4 was separated into “F43 Neurotic Adjustment” representing reactions to extrinsic circumstances and “F4x Neurotic Anxiety and others” representing rather intrinsic factors. The groups “F2 Delusional”, “F5 Behavioral”, “F6 Personality” were not further divided due to low case counts. The value true was assigned to a case if at least one of the underlying ICD-10-codes for each respective mental comorbidity subgroup was recorded as a secondary diagnosis.

*Feature variables: Somatic comorbidity.* The number of comorbid somatic ICD-10 codes was determined by counting the number of codes that were reported as secondary diagnosis and did not belong to ICD-10 Chapter V, as stated above. There exist multiple options to map ICD-10 codes to meaningful groups. The comorbidity indices Elixhauser Score and Charlson Comorbidity Index were developed mainly for the context of mortality and result in rather broad groups [35]. PheCodes were developed in the context of genome-wide association studies and result in very specific groups [36]. World Health Organisation (WHO) morbidity and mortality lists were created for the purpose of national and international reporting of epidemiology and therefore were chosen for this study [37]. The 298 WHO categories were mapped to 35 broader categories, of which 30 were used as somatic features. See Supplementary material 1 in Additional File 2 for a detailed description of the derivation. For each of the 30 somatic comorbidity categories, the value true was assigned to a case if at least one of the underlying ICD-10-codes was recorded as a secondary diagnosis.

### Analysis

*Data Splitting.* For each department, the data was split into a train (80%) and a test set (20%) at random. The test set served the purpose of unbiased model evaluation while the train set was used for all preprocessing steps and training the models.

*Descriptive statistics* were computed by department and data split. Variables are designated either target (what to predict) or features (what may influence the prediction). For continuous variables, the mean, standard deviation, and range were computed, while for categorical variables the ratio within the respective department and data split was reported.

*Odds ratio prolonged length-of-stay and mental comorbidity.* As mental comorbidity was of major interest in this study, the relationship between prolonged length-of-stay and mental comorbidity presence was first investigated by computing odds ratios for each department with 95% confidence intervals. This was computed to highlight the need for separate prediction models for the departments.

**Fig. 1:**
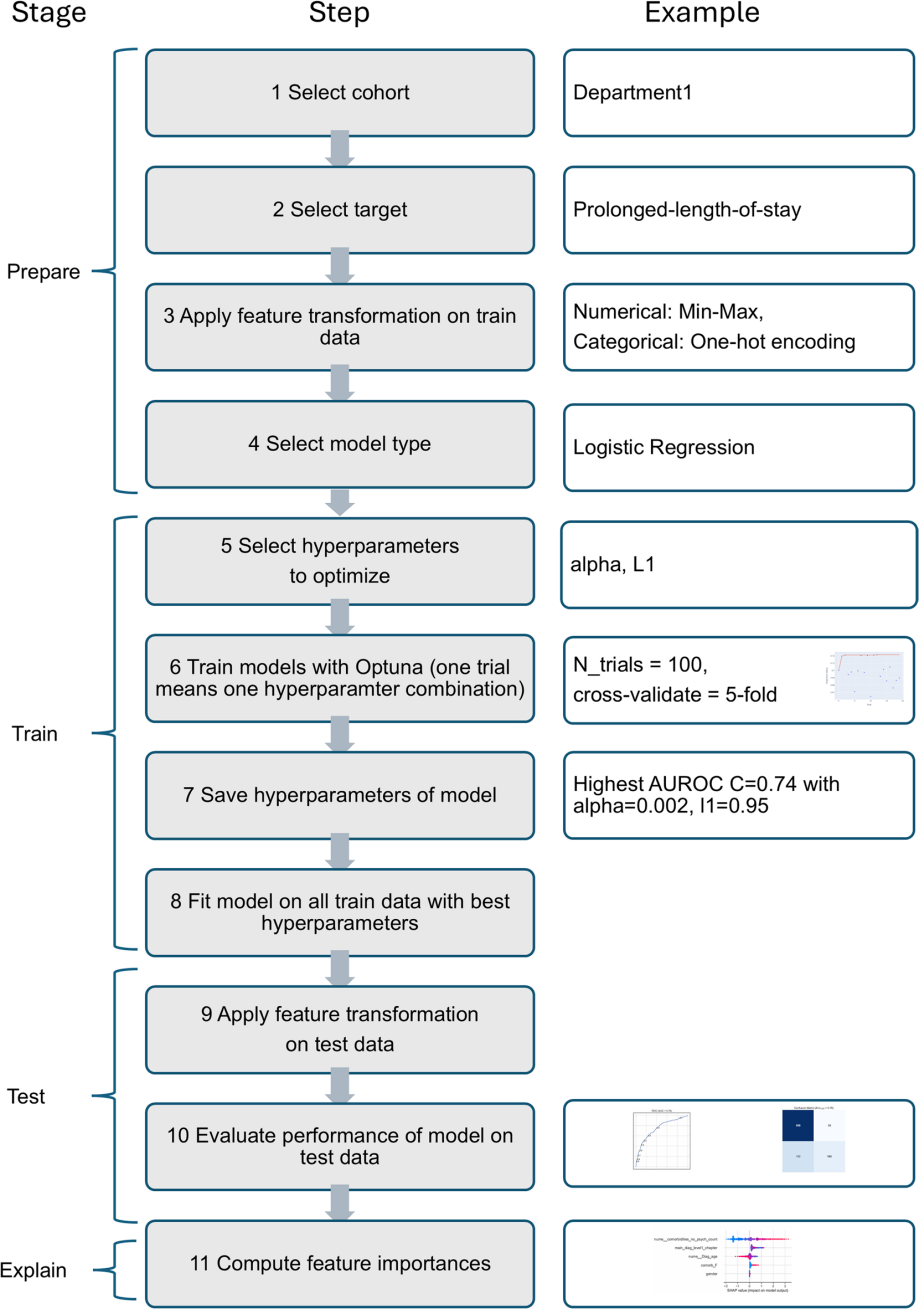
Analysis process exemplified for one model. AUROC: Area under the receiver operator curve

*Classification models*. Figure 1 shows the sequence of steps during the four stages of the analysis process. The first stage involved preparatory steps to select varying factors, such as department, target, feature transformations (MinMaxScaler for numerical variables, based solely on information present in the training data to avoid data leakage [38]) and model type. The outcome target was prolonged length-of-stay (plos). Four different supervised machine learning algorithms were implemented: Elastic-Net Logistic Regression (LR), Random Forest Classifier (RF), Extreme Gradient Boost (XGB), Light Gradient Boosting Machine (LGBM). Tree-based models are suited especially well for tabular data, elaborate discussion on algorithms and performance measure in supplementary material of Bopche et al. [17].

During the second stage, on the train data only, we conducted automatic hyperparameter optimization with the Optuna framework [21, 39, 40]. Optuna chooses a new combination of hyperparameter values during each so-called trial. The number of trials was set to 100. Within each of the 100 trials, 5-fold cross-validation took place. This means that the train data was again split 5 times into 20% validation set and 80% train set. The suggested ranges to start the hyperparameter search from for each model type are in the code repository (10.11588/DATA/HP9O2J). The scoring parameter in the objective to optimize was the area under the receiver operating characteristic curve (AUROC). AUROC has the advantage that it is suited for imbalanced classes and considers varying classification thresholds [21]. An AUROC of 0.5 would mean the model is no better than random guessing, a value of 1.0 means perfect discrimination. AUROC ranges in between can be roughly categorized as acceptable ≥ 0.7, good ≥ 0.8, or excellent ≥ 0.9 [15, 16]. The mean AUROC of the 5-fold cross-validation was the reported performance value for the specific hyperparameter combination of the respective trial. The hyperparameters among all 100 Optuna trials that led to the highest AUROC were used to compute a final model for each model type fitted with the complete train data.

In the third stage, this final model was used to make predictions on the test data to yield an unbiased model evaluation for each model type. To evaluate model performance on test data, the following outcome measures were recorded as assessments of discrimination power: AUROC, accuracy, recall, specificity, balanced accuracy, precision, weighted precision, F1-score, and weighted average precision score (approximation of weighted area under the precision-recall-curve). The Brier score was computed as a measure for calibration, lower values meaning better calibration [41]. If the study’s objective had been to choose the best model type or to build a high-performing ensemble model [42], e.g. for application in clinical routine, another test data set would be required after this step to evaluate performance on unseen data with only the model type that performed best by a pre-defined measure. As the aim of this study was, however, focused on establishing a baseline for understanding the relative importance of comorbidity features, this study’s framework stopped model evaluation at this phase.

In the last stage, explaining the model via SHapley Additive exPlanations (SHAP) values was explored. SHAP values are based on a model-agnostic way to compute feature importance that analyzes the change in the expected model prediction when conditioning on that feature [43]. SHAP values were computed for each individual case and then aggregated as the mean absolute value in both the training and test data separately. To compare the rankings of the features across departments, stacked ranking charts were constructed for the models with the highest AUROC [27]. The visualization method was enhanced by incorporating also the numerical SHAP values and providing the output as a sortable excel file. The code for constructing this visualization is provided in the supplement repository (10.11588/DATA/HP9O2J) and can be applied to a broad set of situations where ranks of features are to be compared.

The implementation of the classification model training, evaluation and result reporting involved the python packages pandas 1.3.4 [44, 45], optuna 3.6.1 [39], scikit-learn 1.5.0 [46], numpy 1.26.4 [47], matplotlib 3.5.0 [48], shap 0.45.1 [43], and openpyxl 3.0.9 [49].

## Results

### Sample description

Cases were 35–50% female and mean age at admission ranged from 56 to 70. Additional File 1 shows the characteristics of the study participants by department and data split. Regarding the threshold for prolonged length-of-stay by department, it was considered prolonged when the number of days at the hospital was greater than or equal to 9 (Cardiology, median 5 days), 10 (General and Psychosomatics, Gastroenterology, Medical Oncology, median 6 days), 11 (Endocrinology, median 7 days) and 26 (Hematology, median 14 days). Refer to Additional File 2 Supplementary material 2 for histograms of the length-of-stay distribution for each department and Supplementary material 3 for how these values relate to the 70th -85th length-of-stay percentiles. The fraction of prolonged length-of-stay cases ranged from 19.3 to 30.6%. The most frequent main diagnoses in Endocrinology varied widely with the prominent chapters being IV Endocrine, I Infectious, and XIV Genitourinary. In General and Psychosomatics, and Cardiology more than 80% had main diagnoses from chapter IX Circulatory. XI Digestive made up 56% in Gastroenterology, followed by 12% II Neoplasms. For Hematology and Medical Oncology more than 70% had II Neoplasms.

The fraction of cases with mental comorbidity across departments ranged from 5.4 to 20.4% in the training sets (total cohort 15.2%, refer to Additional file 1). Overall, the most frequent mental comorbidity subgroups were F05 Organic Delirium with 3.4%, F17 Substance Tobacco with 3.0% and F43 Neurotic Adjustment with 2.8% of the total study. The number of somatic comorbidities ranged from an average of 5.5 to 14.7. The most frequent somatic comorbidity subgroups were Endocrine, nutritional and metabolic with 55.6%, Hypertensive 50.1% and Circulatory system 46.1% of the total study population. Across departments, the prevalences of the subgroups were very heterogeneous.

### Odds ratio prolonged length-of-stay and mental comorbidity

For each department, count and percentage of the total cases as well as counts and department-level percentages for prolonged cases and cases with mental comorbidity are shown in Table 2. In addition, the odds ratio for having prolonged length-of-stay with mental comorbidity in comparison to no mental comorbidity is reported. For cases of department General and Psychosomatics, the odds of prolonged length-of-stay were only 1.27-fold higher with than without mental comorbidity and this difference was not significant. The cases in the other departments had 2.11 to 4.4-fold higher odds of having prolonged length-of-stay given mental comorbidity than given no mental comorbidity. These differences support the need to compute separate prolonged length-of-stay prediction models for each department, as varying predictive power can be expected.

**Table 2:** Descriptive statistics and odds ratio for prolonged length-of-stay with and without mental comorbidity by department. CI: confidence interval

Department	Pro-longed length-of-stay threshold in days	Patients total *N*	Cases department total *N*	Cases % ot cohort total	Cases pro-longed *N*	Cases pro-longed % of depart-ment total	Cases with mental co-morbidity *N*	Cases with mental co-morbidity % of depart-ment total	Odds ratio	Odds ratio 95% CI	Odds ratio *p*-value
1 Endocrinology	≥ 11	3746	4355	15.26%	1281	29.41%	570	13.09%	2.99	[2.49, 3.60]	1.80E-32
2 General and Psychosomatics	≥ 10	2165	2316	8.12%	479	20.68%	433	18.70%	1.27	[0.98, 1.64]	0.07
3 Cardiology	≥ 9	9748	12,056	42.25%	3408	28.27%	1927	15.98%	2.11	[1.91, 2.34]	2.09E-46
4 Gastro-enterology	≥ 10	3560	5115	17.92%	1558	30.46%	1045	20.43%	2.43	[2.11, 2.80]	9.51E-35
5 Hematology	≥ 26	1627	3232	11.33%	631	19.52%	176	5.45%	4.4	[3.19, 6.07]	1.39E-19
6 Medical Oncology	≥ 10	916	1462	5.12%	369	25.24%	173	11.83%	3.23	[2.30, 4.54]	5.54E-12

### Classification models prolonged length-of-stay

Table 3 shows the results for all models predicting prolonged length-of-stay. The AUROC varied only marginally among model types. The prediction performance in terms of AUROC was lowest for department General and Psychosomatics (0.7194), and highest for Hematology (0.8868). Hematology also had the highest accuracy with 85.78% of test cases predicted correctly (in comparison to 79.2% probability of guessing correctly the non-prolonged cases). The hyperparameters that were used to build the models with highest AUROC are listed in Supplementary material 4 in Additional File 2.

**Table 3:** Performance measures of all models computed for prolonged length-of-stay prediction on test data. Models with highest AUROC for each department are marked bold. AUROC: area under the receiver operator curve

Department title	Model type	AUROC	Accuracy	Balanced Accuracy	Precision	Weighted Precision	Recall	Specificity	F1-Score	Weighted Average Precision Score	Brier
**1 Endocrinology**	**XGBoost**	**0.7616**	**0.7635**	**0.6617**	**0.6463**	**0.7486**	**0.4173**	**0.9060**	**0.5072**	**0.4397**	**0.1666**
	Random Forest	0.7599	0.7658	0.6633	0.6543	0.7513	0.4173	0.9092	0.5096	0.4430	0.1642
	LightGBM	0.7565	0.7646	0.6625	0.6503	0.7499	0.4173	0.9076	0.5084	0.4413	0.1649
	Logistic Regression	0.7505	0.7532	0.6428	0.6275	0.7355	0.3780	0.9076	0.4717	0.4185	0.1703
**2 General and Psychosomatics**	**Random Forest**	**0.7194**	**0.8017**	**0.5431**	**0.5882**	**0.7645**	**0.1053**	**0.9810**	**0.1786**	**0.2451**	**0.1453**
	XGBoost	0.7130	0.7996	0.5496	0.5455	0.7576	0.1263	0.9729	0.2051	0.2478	0.1466
	LightGBM	0.7095	0.8082	0.5863	0.5882	0.7770	0.2105	0.9621	0.3101	0.2855	0.1468
	Logistic Regression	0.7016	0.7931	0.6315	0.4928	0.7733	0.3579	0.9051	0.4146	0.3078	0.1547
**3 Cardiology**	**XGBoost**	**0.7978**	**0.7877**	**0.6734**	**0.7028**	**0.7761**	**0.4146**	**0.9321**	**0.5215**	**0.4547**	**0.1489**
	Random Forest	0.7958	0.7902	0.6723	0.7203	0.7801	0.4056	0.9390	0.5190	0.4580	0.1495
	LightGBM	0.7953	0.7869	0.6741	0.6963	0.7748	0.4190	0.9293	0.5232	0.4539	0.1486
	Logistic Regression	0.7867	0.7902	0.6710	0.7239	0.7805	0.4012	0.9408	0.5163	0.4575	0.1502
**4 Gastro-enterology**	**Random Forest**	**0.7883**	**0.7908**	**0.6905**	**0.7627**	**0.7865**	**0.4397**	**0.9413**	**0.5579**	**0.5035**	**0.1559**
	XGBoost	0.7845	0.7830	0.6840	0.7322	0.7755	0.4365	0.9316	0.5469	0.4887	0.1553
	Logistic Regression	0.7824	0.7771	0.6808	0.7068	0.7673	0.4397	0.9218	0.5422	0.4789	0.1556
	LightGBM	0.7791	0.7781	0.6768	0.7222	0.7697	0.4235	0.9302	0.5339	0.4788	0.1567
**5 Hematology**	**Random Forest**	**0.8868**	**0.8578**	**0.6959**	**0.7200**	**0.8458**	**0.4320**	**0.9598**	**0.5400**	**0.4208**	**0.1014**
	Logistic Regression	0.8842	0.8532	0.6687	0.7419	0.8412	0.3680	0.9693	0.4920	0.3951	0.1043
	XGBoost	0.8827	0.8578	0.7172	0.6854	0.8467	0.4880	0.9464	0.5701	0.4334	0.0999
	LightGBM	0.8800	0.8485	0.7053	0.6484	0.8363	0.4720	0.9387	0.5463	0.4080	0.1011
**6 Medical Oncology**	**Logistic Regression**	**0.8227**	**0.7918**	**0.7133**	**0.6923**	**0.7826**	**0.5233**	**0.9034**	**0.5960**	**0.5022**	**0.1467**
	XGBoost	0.8145	0.7986	0.7046	0.7455	0.7917	0.4767	0.9324	0.5816	0.5090	0.1496
	Random Forest	0.8101	0.7952	0.6919	0.7600	0.7900	0.4419	0.9420	0.5588	0.4996	0.1547
	LightGBM	0.8085	0.8020	0.7070	0.7593	0.7963	0.4767	0.9372	0.5857	0.5156	0.1514

**Fig. 2:**
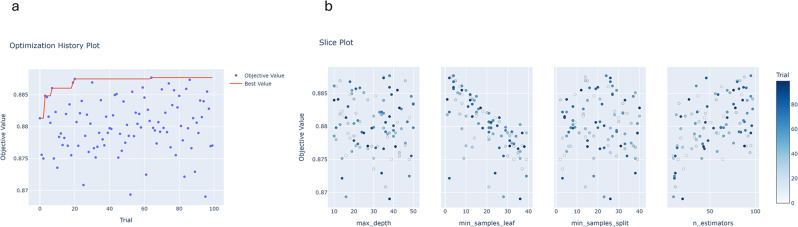
Optuna trial history for 100 hyperparameter combinations that yielded the hyperparameters used for fitting the final prediction model for prolonged length-of-stay (≥ 26 days) for department 5 Hematology with Random Forest. **a**) Optimization History; **b**) Slice plot for each hyperparameter. max_depth: maximum depth of the tree, min_samples_leaf: minimum number of samples required to be in a leaf node, min_samples_split: minimum number of samples required to split an internal node, n_estimators: number of trees in the forest

The results available for all models are now exemplified by the overall best performing model Random Forest in Hematology. The Optuna optimization history that was the underlying mechanism for determining the hyperparameters for this model is displayed in Fig. 2. The best objective value with AUROC = 0.888 was achieved with the hyperparameter combination of trial number 64. The slice plots for each varying hyperparameter show that better results were achieved with smaller values for min_samples_leaf and at least 25 n_estimators, while max_depth and min_samples_split did not show particular patterns. These observations match the hyperparameters chosen by Optuna for fitting the final model: ‘n_estimators’=95, ‘max_depth’=38, ‘min_samples_split’=24, ‘min_samples_leaf’=4.

**Fig. 3:**
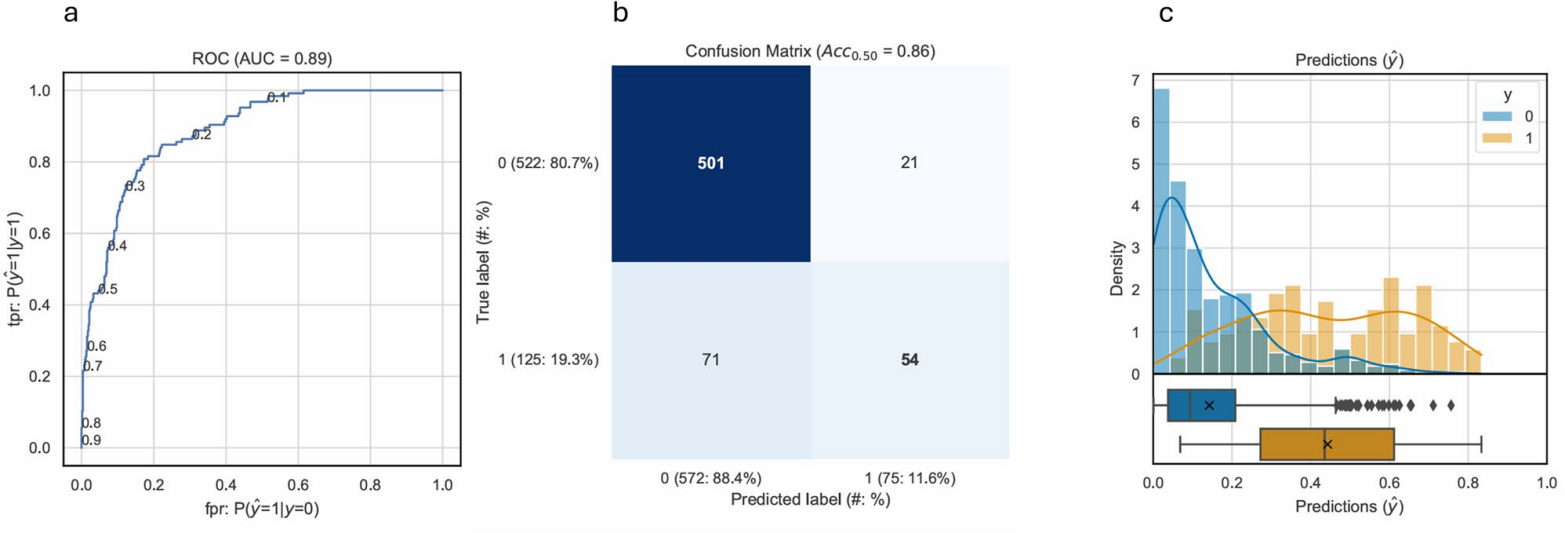
Performance of prediction model for prolonged length-of-stay (≥ 26 days) for department 5 Hematology with Random Forest. **a**) ROC: Receiver Operator Curve, AUC: AUROC = Area under the Receiver Operator Curve; **b**) Confusion Matrix, ACC: Accuracy; c) Histogram of predicted probabilities colored by true values

Figure 3 visualizes several performance metrics for this final model. The Receiver Operator Curve (ROC) is the curve on which the AUROC is based. The confusion matrix shows exact counts of true and false positives and negatives in the test data. The Predictions plot shows the distribution of true values among the predicted probabilities. The truly prolonged cases were predicted more often as false negatives (*N* = 71 false negatives and 54 true positives), demonstrated by the slight shift of the yellow box plot to the left of the predicted class attribution threshold 0.5.

**Fig. 4:**
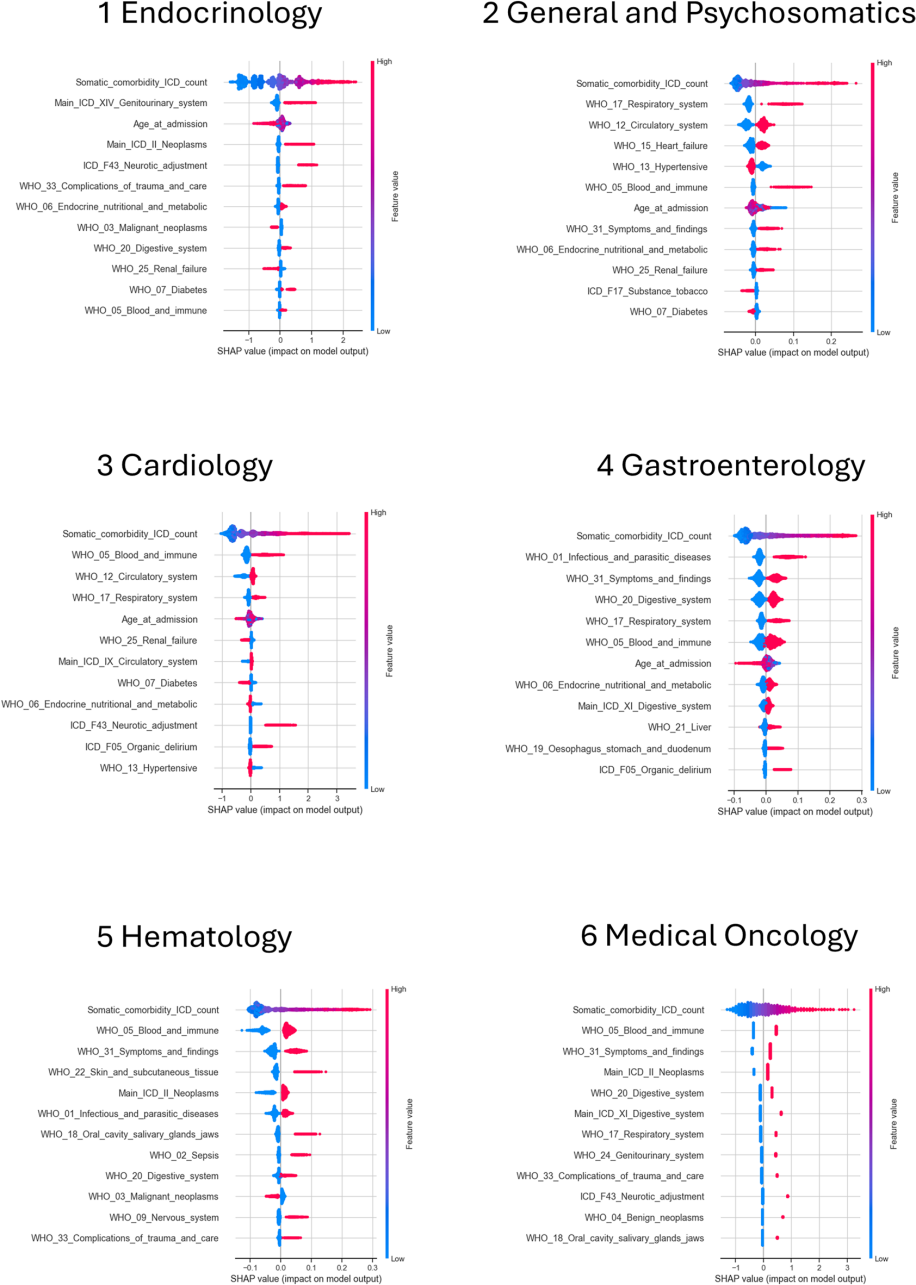
Beeswarm plots of top 12 features predicting prolonged length-of-stay ranked by mean absolute SHAP value, computed on train data. Displayed are models with highest AUROC per department, x-axis scale varies depending on model type. Red color for categorical variables referred to the value “true”

**Fig. 5:**
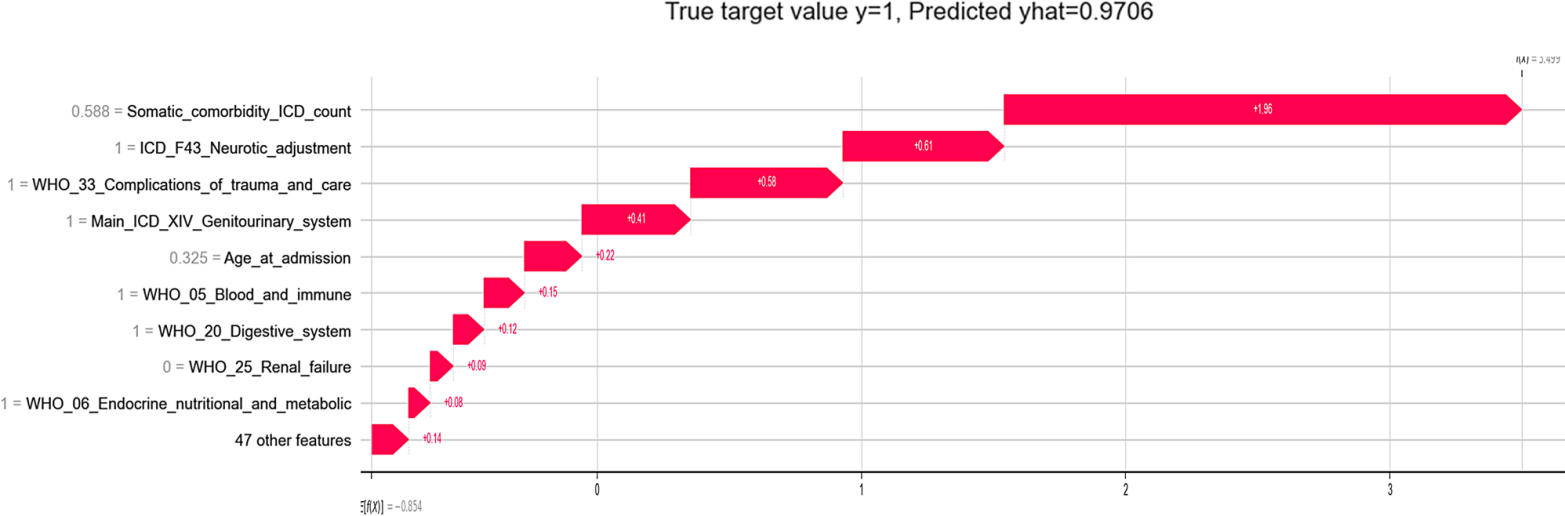
SHAP values for an individual case. This case is from the training data of the Endocrinology department with a main diagnosis in ICD-10 chapter XIV genitourinary system. It was correctly classified by the XGB model as prolonged with high certainty of the model (predicted yhat = 0.9706 is close to true target value y = 1). This case serves only as an illustration of how Fig. 4 was constructed and as an example of the way that individual predictions could be presented for actionable clinical decisions with more accurate underlying models and is not to be interpreted directly

For the most important features, the SHAP values for all cases of the train data were plotted as points in Fig. 4. The results of the train data were chosen for the detailed report to fulfill the aim of understanding which features were important from the angle of what the models learned during the training stage. Each case is represented as a color-coded point for each feature. Here, not only the Random Forest of Hematology is shown, but for all departments the rankings of features by their mean absolute SHAP values in the best performing models. To exemplify how the SHAP values for a single case are typically presented, one of the Endocrinology cases of the training data is shown in Fig. 5. For this case, the highest contributions to the classification as prolonged were the high number of somatic ICD-10 codes and comorbid adjustment disorder.

The mean absolute SHAP values across all train data cases are reported in a sortable excel file in Additional File 3 up to rank 30. The top 5 ranking features will be named here for each department. The somatic comorbidity ICD-10 count was ranking highest in all models. For Endocrinology cases, it was followed by a main diagnosis of the genitourinary system, the age at admission, main diagnosis neoplasms and comorbid adjustment disorder. For General and Psychosomatics cases, comorbidity of the respiratory system, circulatory system, heart failure and hypertensive disease ranked next. Length-of-stay for Cardiology cases was most influenced by diseases of the blood and immune system, circulatory system, respiratory system and age at admission. For Gastroenterology cases, the influence was greatest by infectious and parasitic diseases, general symptoms and findings, digestive system and respiratory system disorders. Both Oncology departments had blood and immune system, general symptoms and findings and the main diagnosis neoplasms among the most influential factors. For Hematology cases, also skin and subcutaneous tissue diseases were ranked high, and the digestive system for Medical Oncology.

## Discussion

In contrast to a previous publication using the same data source [6], the approach described within this study treated length-of-stay as a classification instead of a regression problem, followed a machine learning methodology instead of a single model fit, treated each department as a separate sub-cohort, and modeled mental and somatic comorbidities on a more detailed level.

### Implications of results

To our knowledge, this is the first study to report the prolonged length-of-stay threshold department-wise for internal medicine, to use the median absolute deviation as a distribution-suitable method to determine the threshold as an outlier and to enable interpretation of prolonged length-of-stay prediction on a detailed diagnosis level for somatic as well as mental comorbidities. We reported machine learning models for predicting prolonged length-of-stay for six internal medicine departments and the most impactful variables. Determining the thresholds for prolonged length-of-stay on a department-level yielded more than or equaling 9 days for Cardiology, 10 days for General and Psychosomatics, Gastroenterology, Medical Oncology, 11 days for Endocrinology and 26 days for Hematology. This means for the department Hematology, if the threshold had been chosen between 9 and 11 days, as for the other departments, most cases would have been classified as prolonged even though a longer length-of-stay appeared to be expected with the median at 14 days, which aligns with a Canadian study on hematologic oncology inpatients requiring rapid response system activation that found a median hospital length of stay of 19 days for the entire cohort (*n* = 401) and 26.5 days for the subgroup (*n* = 145) that received hematopoietic cell transplants (HCT) [50]. The multi-modal length-of-stay distribution may relate to those unique characteristics of hematologic oncology patients incurred by the high risk of infection due to immunosuppression and the various treatment regimens such as HCT [50] and should be subject of further investigation. The machine learning models’ performance measured as best AUROC per department was in the range of 72 to 89%. The most impactful variables included the number of somatic ICD-10 codes, the age at diagnosis, and multiple mental and somatic comorbidity subgroups.

Previous studies reported discriminatory power for predicting prolonged length-of-stay in terms of AUROC ranging from 75% [16] to 98% [17]. The models in the presented study with AUROC from 72 to 89% were in a similar range. The low odds ratio for mental comorbidity and prolonged length-of-stay at the department for General and Psychosomatics already lowered the expectation for possible predictive power of the models. This may be explained by the strong focus on psychosomatics at this department, where treatment of mental comorbidities is already integrated in resource planning while the prolonging effect of additional mental burden is higher at the other departments. But this means that also the other somatic features apparently did not suffice as good predictors for predicting prolonged length-of-stay and factors not in the dataset may be responsible for length-of-stay outliers here, suggesting further data sources or transformations to be explored. Across all departments, the performances of the prolonged length-of-stay prediction models were acceptable enough to explore the aggregation of the most important features, but not yet accurate enough to apply the models to individual case predictions as very high confidence in the prediction results would be required for that application [15, 16].

This kind of discussion of the aggregate importance of features for our study is attempted in more detail here for the top 12 features by mean absolute SHAP values. The visual realization of the SHAP value distribution and ranking serve in the discussion about ranks and direction of feature impact on prolonged length-of-stay prediction. Across all departments, the over-proportionally large SHAP values of somatic comorbidity ICD-10 count were probably due to the numerical nature of this feature, while all other comorbidity features were represented categorically. Nonetheless, the somatic comorbidity ICD-10 count was included, as it has been reported as an important predictor for prolonged length-of-stay before, which our study confirmed [51]. However, the minimum-maximum scaling (scikit-learn MinMaxScaler) probably was not optimal for the long-tailed right-skewed distribution of somatic comorbidity ICD-10 count ranging up to 94 codes and other ways to transform this feature should be explored in the future, such as RobustScaler or QuantileTransformer [46]. Especially when generalizability is to be tested by using various institutions’ datasets, the transformation of the number of diagnosis codes needs to be reconsidered, as different limits exist in the number of codes allowed in the electronic health records of the patients across health systems (e.g. 20 codes in England, 14 codes in Wales, 6 codes in Scotland for UK Biobank data [52]). And if the high SHAP values stem from the simple interpretation that the number of diagnosis codes is indeed an important predictor for length-of-stay, the consequences of putting potentially arbitrary technical limitations on health record databases should be considered with subsequent research in mind. Age at admission was the only other numerical variable and appeared four times in the top 12. Younger age was associated with longer length-of-stay, as previously reported [6].

Among the binary variables pertaining to detailed diagnosis groups, especially those that are not directly related to the focus area of the department are of interest. Both mental and somatic comorbidities appeared in the most important factors influencing prolonged length-of-stay. For mental comorbidities, Neurotic, stress-related and somatoform disorders (F43) appeared three times in the top 12, at Endocrinology, Cardiology and Medical Oncology. The importance of adjustment disorders for length-of-stay suggests the hypothesis that psychological processing of the consequences of the somatic main diagnosis can be regarded as direct result of the somatic disease. The relationship of mental disorders as a kind of psychological processing related to diagnosis with diabetes, cardiovascular disease or cancer has been indicated before. At Endocrinology, many admissions involve diabetes and hormonal disorders. For diabetes, stressors in the form of disease management and fear of or already occurring somatic complications were described to have debilitating emotional impact that in turn make carrying out diabetes care tasks more difficult [53]. At Cardiology, patients may have just faced a sudden life-threatening situation in the form of a cardiac event. High prevalence of stress-response and affective disorders among cardiac patients [54, 55] and posttraumatic stress disorder as a consequence of acute cardiovascular disease have previously been reported [56]. At Oncology, cancer diagnoses impose great challenges for the patients’ physical as well as psychological well-being and the strong connection gave rise to the field of psycho-oncology [57]. At least 30–35% of oncology patients are affected by psychiatric disorders, even though the clear attribution of symptoms to specific diagnoses remains difficult, as e.g. depression, stress-related disorders and somatoform disorders have overlap [58].

Delirium as part of organic mental disorders appeared in the top features at Cardiology and Gastroenterology. This finding matches a review of multiple studies that report delirium in critical illness to be independently predictive of excess hospital length-of-stay [59]. Substance abuse of tobacco was associated with reduced probability of prolonged length-of-stay for General and Psychosomatics. A study on elective surgeries reports the opposite direction, with longer length-of-stay for smokers in comparison to non-smokers [60]. The discrepancy might be explained by the difference in methods, as our study was for predicting more extreme outliers, while Arena et al. [60] compared mean length-of-stay for non-smokers and smokers. One hypothesis for explaining our finding could be that the diagnosis of substance abuse tobacco indicates strong addiction behavior with increased motivation to leave the hospital in order to resume tobacco consumption in a non-hospital environment.

Somatic comorbidities not directly related to department focus areas included diagnoses such as complications of trauma and care or comorbid blood and immune system disorders. Symptoms and findings not elsewhere classified appeared four times in the top 12, and further investigation showed that the top detailed diagnoses counted in this variable included systemic inflammatory response syndrome (SIRS), shock (cardiogenic, hypovolemic, septic) and abnormalities of breathing. These rather systemic medical issues seem intuitive to raise length-of-stay predictions as they are connected to symptoms that affect the entire body [37]. The more organ-specific complication renal failure appeared three times in the top 12. Respiratory, genitourinary, circulatory and endocrine and metabolic diseases were among the top somatic comorbidity features in our study as well as in a study that focused on patients suspected of bloodstream infections [17].

### Limitations and outlook

Limitations of this study and ideas for future work are discussed along the numerous decisions on the methods of modeling length-of-stay with comorbidities as predictors.

The first decision involved cohort selection. The study analyzed cases treated at the Center of Internal Medicine of the University Hospital in Heidelberg. Even though the entire ICD-10 spectrum of internal diseases was covered by the cohort, ensuring representativity of a broad range of diseases, the case constellations represent a maximum-care university hospital, which is a specific case of the main and maximum care tier of the German four-tier hospital structure next to primary care, standard care and specialized hospitals [61]. In addition, two departments were part of other overarching central structures and allowed for highly specialized clinical teams. We aimed to follow the unified framework for LoS prediction [1], but the generalization to other study populations from different settings was not yet implemented, and should definitely be considered by future research to investigate how representative of similar and other hospital types the findings are. Our study pointed out considerations to keep in mind when feature engineering is planned for testing generalizability across hospital systems or countries, such as a broadly applicable way of rescaling numerical variables that is independent of locally enforced data ranges as much as possible, such as for the number of diagnosis codes allowed in electronic health records. The concept of department-wise considerations should be comparable to other institutions, as similar hospital clinic structures and department sub-structures can be expected to exist nationally and internationally. As observations based on cases are not completely independent of each other due to rehospitalizations, patient-level analyses should be considered in the future.

The second decision was the target to be predicted. It is notable that the MAD-based thresholds for prolonged length-of-stay lay between the 70th -85th percentiles of the underlying populations for all departments, which aligned with the 75th percentile approximation used in several previous studies. This may relate to the specific choices of scaling parameters applied to the MAD itself. If the usage of MAD is scaled by an additional 2.5 or 3-fold [14], higher class imbalance is incurred and the research aim would target very extreme outliers. Instead of treating prolonged length-of-stay as a binary classification problem to detect outliers, inspiration can be gained from its perception as a multi-class classification, where the division across several thresholds is chosen instead of a single threshold [20, 30]. A multi-class approach provides more perspectives on the concept of a length-of-stay outlier, however, entails even more degrees of freedom of the thresholds’ to be justified. Modeling length-of-stay as a regression problem has the merit of a numerical prediction output with more nuances of the distribution accounted for, but the drawback of choosing a comparable reporting performance measure. Also, the discussion of which threshold constitutes an alarmingly high length-of-stay still has to be held by the users of a regression model prediction output. Another approach could involve time-to-event analysis [62]. A comparison of trade-offs of common length-of-stay modeling approaches was conducted by Muhlestein et al. [42].

The third decision regarded the potential influencing factors on length-of-stay. The information employed by this study, age, gender, primary diagnosis and comorbidity, were among the key features that appeared in multiple length-of-stay studies [1], however, information on potential confounders such as disease severity as well as social and economic status was missing. Especially for General and Psychosomatics patients, incorporation of patient-specific factors, such as personality functioning [63], chronification [64] and pre-therapies may have added boosts to prediction performance. Another aspect of potentially not-covered information is the collection of clinically relevant psychosocial dimensions, not detectable by using the classical Diagnostic and Statistical Manual of Mental Disorders (DSM) and ICD psychiatric nosological systems that was proposed as the Diagnostic Criteria for Psychosomatic Research (DCPR) [65, 66]. The DCPR dimensions include a series of syndromes, such as demoralization (detectable in about one-third of medically ill patients), irritability, and alexithymia [65]. Rajkomar et al. demonstrated the potential of comprehensive electronic health record data including laboratory values, medication and unstructured data in a study that achieved an AUROC of 86% with a free text based approach on a large cohort for prediction of length-of-stay ≥ 7 days [5]. The tokens derived from free text were not reported as aggregated importance, however, but only a case study showed which tokens were used for the prediction for one case. The authors emphasized that more research was necessary regarding applicability across predictions and clinical utility.

The fourth decision was manifold along the model building and evaluation process. The overall modest performance of the models could probably be improved by more extensive tuning of hyperparameters, techniques to handle class imbalance [17] and other feature transformations, such as ways of counting or grouping comorbidities. The objective was not to generate models that were as performant as possible, but to gain a first impression of the ranking of mental and somatic comorbidity influence on prolonged length-of-stay. Knowledge about the most relevant comorbidities for length-of stay could be used for adaptive bed capacity planning when patients are predicted to stay longer based on comorbidity profiles. To achieve this kind of applicability in a real-time clinical setting would, however, require models that are accurate enough to allow individual case predictions and the early availability of the diagnostic information during the hospital stay.

## Conclusions

Taking department-specific factors into account is advisable for analyses of length-of-stay, especially if a threshold for distinguishing prolonged outliers is necessary. We recommend for future studies to expand on this approach to report a reproducible mapping of ICD-10 codes to model comorbidities in a comparable categorization into consistent groups. Machine learning models trained with comorbidities represented as ICD-10 code based features can deliver aggregated insights, however, are not yet performant enough for actionable individual case predictions. Mental as well as somatic comorbidities belonged to the most relevant variables for the prediction of prolonged length-of-stay. Only if mental comorbidities are incorporated in hospital quality of care research, insurances can be informed about the diagnosis combinations that are expected to draw more resources to motivate adequate reimbursement and investments in preventive measures.

## Supplementary Information

Below is the link to the electronic supplementary material.

Supplementary Material 1

Supplementary Material 2

Supplementary Material 3

Supplementary Material 4

Supplementary Material 5

## Data Availability

Data cannot be shared publicly because of personal patient data gathered in clinical routine that underlies personal data protection regulations. Data may be available (contact via corresponding author) for researchers who meet the criteria for access to confidential data. Materials in the form of code used for the analysis are published in the following repository: 10.11588/DATA/HP9O2J.

## Abbreviations

ACC: Accuracy
AUROC: Area under the receiver operator curve
ICD-10: (International Statistical Classification Of Diseases And Related Health Problems, 10th revision, German Modification)
ICD_F: F-chapter for Mental disorders and behavioral disorders of the ICD-10
LIME: Local Interpretable Model-agnostic Explanations
LGBM: Light Gradient Boosting Machine
LOS: Length-of-stay
LR: Elastic-Net Logistic Regression
MAD: Median Absolute Deviation
PLOS: Prolonged length-of-stay
RF: Random Forest Classifier
RNN: Recurrent neural network
ROC: Receiver Operator Curve
SHAP: Shapley additive explanations
WHO: World Health Organization
XGB: Extreme Gradient Boost

## References

[1] Stone K, Zwiggelaar R, Jones P. Mac Parthaláin, N. A systematic review of the prediction of hospital length of stay: towards a unified framework. PLOS Digit Health. 2022;1:e0000017. 10.1371/journal.pdig.0000017. 36812502 10.1371/journal.pdig.0000017 PMC9931263

[2] Gokhale S, et al. Hospital length of stay prediction tools for all hospital admissions and general medicine populations: systematic review and meta-analysis. Front Med. 2023;10:1192969. 10.3389/fmed.2023.1192969. 10.3389/fmed.2023.1192969 PMC10469540 37663657

[3] Brown AE, Press VG, Meltzer DO. Association of health confidence with hospital length of stay and readmission. J Hosp Med. 2024;19:794–801. 38751348 10.1002/jhm.13405 PMC12879544

[4] Webber AA, et al. Psychiatric diagnoses are associated with postoperative disparities in patients undergoing major colorectal operations. Am Surg. 2024;90:2695–702. 38650166 10.1177/00031348241248690

[5] Rajkomar A, et al. Scalable and accurate deep learning with electronic health records. Npj Digit Med. 2018;1. 10.1038/s41746-018-0029-1. 10.1038/s41746-018-0029-1 PMC6550175 31304302

[6] Stahl-Toyota S, et al. Interaction of mental comorbidity and physical Multimorbidity predicts length-of-stay in medical inpatients. PLoS ONE. 2023;18:e0287234. 10.1371/journal.pone.0287234. 37347745 10.1371/journal.pone.0287234 PMC10287009

[7] Bacchi S, et al. Machine learning in the prediction of medical inpatient length of stay. Intern Med J. 2022;52:176–85. 33094899 10.1111/imj.14962

[8] Jaotombo F, et al. Machine-learning prediction for hospital length of stay using a French medico-administrative database. J Mark Access Health Policy. 2022;11:2149318. 10.1080/20016689.2022.2149318. 36457821 10.1080/20016689.2022.2149318 PMC9707380

[9] Goodwin JS, Li S, Kuo Y-F. Association of the work schedules of hospitalists with patient outcomes of hospitalization. JAMA Intern Med. 2020;180:215–22. 31764937 10.1001/jamainternmed.2019.5193 PMC6902197

[10] Golinelli D, Sanmarchi F, Toscano F, Bucci A, Nante N. Analyzing the 20-year declining trend of hospital length-of-stay in European countries with different healthcare systems and reimbursement models. Int J Health Econ Manag. 2024;24:375–92. 38512638 10.1007/s10754-024-09369-0 PMC11457716

[11] Chrusciel J, et al. The prediction of hospital length of stay using unstructured data. BMC Med Inf Decis Mak. 2021;21. 10.1186/s12911-021-01722-4. 10.1186/s12911-021-01722-4 PMC8684269 34922532

[12] Rousseeuw PJ, Croux C. Alternatives to the median absolute deviation. J Am Stat Assoc. 1993;88:1273–83.

[13] Yang J, Rahardja S, Fränti P. Association for Computing Machinery, Outlier detection: how to threshold outlier scores? in Proceedings of the International Conference on Artificial Intelligence, Information Processing and Cloud Computing. 2019;1–6; 10.1145/3371425.3371427.

[14] Leys C, Ley C, Klein O, Bernard P, Licata L. Detecting outliers: do not use standard deviation around the mean, use absolute deviation around the median. J Exp Soc Psychol. 2013;49:764–6.

[15] Jaotombo F, Adorni L, Ghattas B, Boyer L. Finding the best trade-off between performance and interpretability in predicting hospital length of stay using structured and unstructured data. PLoS ONE. 2023;18:e0289795. 10.1371/journal.pone.0289795. 38032876 10.1371/journal.pone.0289795 PMC10688642

[16] Chirongoma T, et al. Predicting prolonged length of hospital stay and identifying risk factors following total ankle arthroplasty: A supervised machine learning methodology. J Foot Ankle Surg. 2024;63:557–61. 38789076 10.1053/j.jfas.2024.05.005

[17] Bopche R, et al. In-hospital mortality, readmission, and prolonged length of stay risk prediction leveraging historical electronic patient records. JAMIA Open. 2024;7:ooae074. 10.1093/jamiaopen/ooae074. 39282081 10.1093/jamiaopen/ooae074 PMC11401612

[18] Shalaby G, et al. Predictors of prolonged hospital stay and in-hospital mortality in female patients with acute myocardial infarction with specific reference to diabetes. Int J Cardiol. 2024;400:131785. 10.1016/j.ijcard.2024.131785. 38242505 10.1016/j.ijcard.2024.131785

[19] Berg AR, et al. Factors associated with unplanned readmissions and prolonged length of stay in patients undergoing primary fusion for congenital scoliosis. Int J Spine Surg. 2024;8614. 10.14444/8614. 10.14444/8614 PMC11483433 39107092

[20] Jain R, Singh M, Rao AR, Garg R. Predicting hospital length of stay using machine learning on a large open health dataset. BMC Health Serv Res. 2024;24:860. 10.1186/s12913-024-11238-y. 39075382 10.1186/s12913-024-11238-y PMC11288104

[21] Karabacak M, Jagtiani P, Shrivastrava RK, Margetis K. Personalized prognosis with machine learning models for predicting in-hospital outcomes following intracranial meningioma resections. World Neurosurg. 2023;182:e210–30. 10.1016/j.wneu.2023.11.081. 38006936 10.1016/j.wneu.2023.11.081

[22] Wang K, et al. A clinical prediction model based on interpretable machine learning algorithms for prolonged hospital stay in acute ischemic stroke patients: a real-world study. Front Endocrinol. 2023;14:1165178. 10.3389/fendo.2023.1165178. 10.3389/fendo.2023.1165178 PMC10703471 38075055

[23] Zhang D, Yin C, Zeng J, Yuan X, Zhang P. Combining structured and unstructured data for predictive models: a deep learning approach. BMC Med Inf Decis Mak. 2020;20:280. 10.1186/s12911-020-01297-6. 10.1186/s12911-020-01297-6 PMC7596962 33121479

[24] Santos G, Ferreira AR, Gonçalves-Pinho M, Freitas A, Fernandes L. The impact of comorbid psychiatric disorders on chronic obstructive pulmonary disease (COPD) hospitalizations: a nationwide retrospective study. Soc Psychiatry Psychiatr Epidemiol. 2024;59:2093–103. 38429541 10.1007/s00127-024-02645-x

[25] Han C, et al. To predict the length of hospital stay after total knee arthroplasty in an orthopedic center in china: the use of machine learning algorithms. Front Surg. 2021;8:606038. 10.3389/fsurg.2021.606038. 33777997 10.3389/fsurg.2021.606038 PMC7990876

[26] Węgiel M, et al. Hospitalization length after myocardial infarction: Risk-Assessment-Based time of hospital discharge vs. Real life practice. J Clin Med. 2018;7:564. 10.3390/jcm7120564. 30567307 10.3390/jcm7120564 PMC6306951

[27] Yasin P, et al. Machine learning-enabled prediction of prolonged length of stay in hospital after surgery for tuberculosis spondylitis patients with unbalanced data: a novel approach using explainable artificial intelligence (XAI). Eur J Med Res. 2024;29:383. 10.1186/s40001-024-01988-0. 39054495 10.1186/s40001-024-01988-0 PMC11270948

[28] Gilbert T, et al. Combining the hospital frailty risk score with the Charlson and elixhauser Multimorbidity indices to identify older patients at risk of poor outcomes in acute care. Med Care. 2023;62:117–24. 38079225 10.1097/MLR.0000000000001962 PMC10773558

[29] Chen R, et al. A study on predicting the length of hospital stay for Chinese patients with ischemic stroke based on the XGBoost algorithm. BMC Med Inf Decis Mak. 2023;23:49. 10.1186/s12911-023-02140-4. 10.1186/s12911-023-02140-4 PMC10031936 36949434

[30] Lai C-H, Mok PK-L, Chau W-W, Law S-W. Application of machine learning models on predicting the length of hospital stay in fragility fracture patients. BMC Med Inf Decis Mak. 2024;24:26. 10.1186/s12911-024-02417-2. 10.1186/s12911-024-02417-2 PMC10826155 38291406

[31] Mittal A, et al. Predicting prolonged length of stay following revision total knee arthroplasty: A National database analysis using machine learning models. Int J Med Informat. 2024;192:105634. 10.1016/j.ijmedinf.2024.105634. 10.1016/j.ijmedinf.2024.105634 39305561

[32] Pham-Gia T, Hung TL. The mean and median absolute deviations. Math Comput Model. 2001;34:921–36.

[33] Wolff J, Heister T, Normann C, Kaier K. Hospital costs associated with psychiatric comorbidities: a retrospective study. BMC Health Serv Res. 2018;18:67. 10.1186/s12913-018-2892-5. 29382387 10.1186/s12913-018-2892-5 PMC5791176

[34] Potter KM, Prendergast NT, Boyd JG. From traditional typing to intelligent insights: A narrative review of directions toward targeted therapies in delirium. Crit Care Med. 2024;52:1285–94. 10.1097/CCM.0000000000006362. 39007569 10.1097/CCM.0000000000006362

[35] Moltó A, Dougados M. Comorbidity indices. Clin Exp Rheumatol. 2014;32:131–4. 25365102

[36] Wu P, et al. Mapping ICD-10 and ICD-10-CM codes to phecodes: workflow development and initial evaluation. JMIR Med Inf. 2019;7:e14325. 10.2196/14325. 10.2196/14325 PMC6911227 31553307

[37] DIMDI. ICD-10-WHO 2019 Regelwerk (Band 2) PDF - Referenzfassung. Deutsches Institut für Medizinische Dokumentation und Information; 2019. https://www.bfarm.de/DE/Kodiersysteme/Services/Downloads/_node.html [last accessed 26 September 2025].

[38] Sasse L, et al. Overview of leakage scenarios in supervised machine learning. J Big Data. 2025;12. 10.1186/s40537-025-01193-8.

[39] Akiba T, Sano S, Yanase T, Ohta T, Koyama M. Optuna: A Next-generation hyperparameter optimization framework. Proc 25th ACM SIGKDD International Conference on Knowledge Discovery & Data Miningc. 2019;2623–2631. 10.1145/3292500.3330701.

[40] Huberts LCE, et al. Predictive analytics for cardiovascular patient readmission and mortality: an explainable approach. Comput Biol Med. 2024;174:108321. 10.1016/j.compbiomed.2024.108321. 38626511 10.1016/j.compbiomed.2024.108321

[41] Luo H, et al. SHAP based predictive modeling for 1 year all-cause readmission risk in elderly heart failure patients: feature selection and model interpretation. Sci Rep. 2024;14:17728. 10.1038/s41598-024-67844-7. 39085442 10.1038/s41598-024-67844-7 PMC11291677

[42] Muhlestein WE, Akagi DS, Davies JM, Chambless LB. Predicting inpatient length of stay after brain tumor surgery: developing machine learning ensembles to improve predictive performance. Neurosurgery. 2018;85:384–93. 10.1093/neuros/nyy343 PMC7137462 30113665

[43] Lundberg SM, Lee S-I. A Unified Approach to Interpreting Model Predictions in NIPS’17: Proceedings of the 31st International Conference on Neural Information Processing Systems. 4768–4777; 10.5555/3295222.3295230 (2017).

[44] McKinney W. Data structures for statistical computing in python in Proceedings of the 9th Python in Science Conference. (eds Stefan van der Walt & Jarrod Millman). 2010;56–61. 10.25080/Majora-92bf1922-00a.

[45] pandas. pandas-dev/pandas: Pandas. The pandas development team, Zenodo, 2020. 10.5281/zenodo.3509134. [last accessed 26 September 2025].

[46] Pedregosa F, et al. Scikit-learn: machine learning in python. J Mach Learn Res. 2011;12:2825–30.

[47] Harris CR, et al. Array programming with numpy. Nature. 2020;585:357–62. 32939066 10.1038/s41586-020-2649-2 PMC7759461

[48] Hunter JD, Matplotlib. A 2D graphics environment. Comput Sci Eng. 2007;9:90–5.

[49] Gazoni E, Clark C. Openpyxl - A Python library to read/write Excel 2010 xlsx/xlsm files, (2024) https://openpyxl.readthedocs.io/en/stable/ [last accessed 26 Sep 2025].

[50] Gershkovich BA-O, et al. Outcomes of hospitalized hematologic oncology patients receiving rapid response system activation for acute deterioration. Crit Care. 2019;23:286. 10.1186/s13054-019-2568-5. 31455376 10.1186/s13054-019-2568-5 PMC6712869

[51] Aubert CE et al. Best Definitions of Multimorbidity to Identify Patients With High Health Care Resource Utilization. Mayo Clin. Proc. Innov. Qual. Outcomes 4;40–49. 10.1016/j.mayocpiqo.2019.09.002 (2020). 10.1016/j.mayocpiqo.2019.09.002 PMC7011007 32055770

[52] UKB. UK biobank hospital inpatient data version 4.0. UK Biobank; 2023. [last accessed 26 Sep 2025].

[53] Tenreiro K, Hatipoglu B. Mind matters: mental health and diabetes management. J Clin Endocrinol Metab. 2025;110:131–6. 10.1210/clinem/dgae607 39998923

[54] Ski CF, et al. Psychological interventions for depression and anxiety in patients with coronary heart disease, heart failure or atrial fibrillation. Cochrane Database Syst Rev. 2024;4. 10.1002/14651858.CD013508.pub3. 10.1002/14651858.CD013508.pub3 PMC10996021 38577875

[55] Bermudez T, et al. The role of daily adjustment disorder, depression and anxiety symptoms for the physical activity of cardiac patients. Psychol Med. 2023;53:5992–6001. 37743836 10.1017/S0033291722003154 PMC10520595

[56] Princip M, Ledermann K. Känel, R. Posttraumatic stress disorder as a consequence of acute cardiovascular disease. Curr Cardiol Rep. 2023;25:455–65. von. 37129760 10.1007/s11886-023-01870-1 PMC10188382

[57] Anghel T, et al. Review of psychological interventions in oncology: current trends and future directions. Medicina. 2025;61:279. 10.3390/medicina61020279. 40005396 10.3390/medicina61020279 PMC11857804

[58] Caruso R, Breitbart W. Mental health care in oncology. Contemporary perspective on the psychosocial burden of cancer and evidence-based interventions. Epidemiol Psychiatr Sci. 2020;29:1–4. 10.1017/S2045796019000866. 10.1017/S2045796019000866 PMC7214708 31915100

[59] Stollings JL, et al. Delirium in critical illness: clinical manifestations, outcomes, and management. Intensive Care Med. 2021;47:1089–103. 34401939 10.1007/s00134-021-06503-1 PMC8366492

[60] Arena G, Cumming C, Lizama N, Mace H, Preen DB. Hospital length of stay and readmission after elective surgery: a comparison of current and former smokers with non-smokers. BMC Health Serv Res. 2024;24:85. 10.1186/s12913-024-10566-3. 38233897 10.1186/s12913-024-10566-3 PMC10792937

[61] Böcken J. Spotlight Healthcare - Reorganizing germany’s hospital landscape. Bertelsmann Stiftung; 2019. [last accessed 17 Oct 2025].

[62] Garg L, McClean S, Meenan BJ, Millard P. Phase-Type survival trees and mixed distribution survival trees for clustering patients’ hospital length of stay. Informatica. 2011;22:57–72.

[63] Dönnhoff I, et al. Predictors for improvement in personality functioning during outpatient psychotherapy: A machine learning approach within a psychodynamic psychotherapy sample. Eur Psychiatry. 2024;67:e79. 39543914 10.1192/j.eurpsy.2024.1780 PMC11730054

[64] Chernew ME. Addressing the chronification of disease. Am J Manag Care. 2017;23:87–8. 28245656

[65] Mattei G, Curatola C, Moscara M. The interplay between Psychiatry, general Practitioners, and other specialists. Comorbidity between mental and physical disorders: Identification, management and treatment. eds. Andrea Fiorillo, Afzal Javed, & Norman Sartorius; Springer Nature Switzerland, 2025. pp. 369–409.

[66] Zipfel S, Löwe B, Giel K, Friederich H-C, Henningsen P. Implementing the biopsychosocial model in clinical medicine: A tribute to Giovanni Fava. Psychother Psychosom. 2023;92:21–6. 36566743 10.1159/000528451

