# Supplementary material for: Machine learning models incorporating somatic and mental comorbidities for prolonged length-of-stay prediction in a maximum care university hospital
Source: BMC Med Inform Decis Mak. 2025 Nov 26;25:436. doi: 10.1186/s12911-025-03290-3 (PMC12670805; doi:10.1186/s12911-025-03290-3)
Supplement: Supplementary file 2 — Supplementary Material 2 [file 12911_2025_3290_MOESM2_ESM.docx]

# Additional File 2

Manuscript: “Machine learning models incorporating somatic and mental comorbidities for prolonged length-of-stay prediction in a maximum care university hospital”

Legend: Supplementary Material.

Supplementary material 1. Details on derivation of WHO comorbidity groups.

Supplementary material 2. a-f: Length-of-stay distribution by department.

Supplementary material 3. Length-of-stay 70th-85th percentiles by department.

Supplementary material 4. Hyperparameters of best models.

Supplementary material 1. Details on derivation of WHO comorbidity groups

The catalogs applied for coding diseases at the time of data collection were the ICD-10-GM (German modification) Version 2019 and Version 2018. The original catalogs were obtained as published by the German Federal Institute for Drugs and Medical Devices (BFRAM: <https://www.bfarm.de/DE/Kodiersysteme/Services/Downloads/_node.html>, accessed 2024-03-12). On download of the codes and their description, morbidity and mortality lists by the World Health Organisation (WHO) are also included, which are to be applied for national and international reporting of epidemiology (ICD-10-WHO 2019 Regelwerk (Band 2) PDF – Referenzfassung, Published by: Deutschen Institut für Medizinische Dokumentation und Information.)

The files used were from the metadata folders: OID 1.2.276.0.76.5.477: ‘icd10gm2019syst_kodes.txt’, ‘morbl_2019.txt’; OID 1.2.276.0.76.5.471: ‘icd10gm2018syst_kodes.txt’, ‘morbl_2018.txt’. From the plain text files, excel file with the headers as described by the metadata for SQL-tables loading were created. These were merged with the supplied WHO morbidity list, which contains 298 categories. The code for merging the ICD-10 catalog with the WHO morbidity list is published in the manuscript code repository (https://doi.org/10.11588/DATA/HP9O2J). It can be used to apply also other kinds of mapping of ICD-10 codes to categories in order to compare the effect of different ways of modeling diagnostic information.

The occurrences of each individual ICD-10 code in the study cohort were counted to determine which detailed diagnoses accounted for the majority of broader categories. Then, the corresponding counts of all occurrences of the 298 categories in the WHO morbidity list were determined. Depending on the number of occurrences and qualitative evaluation of disease differences by ICD-10 blocks or categories, smaller categories were combined into broader categories. The qualitative evaluation also included review of the detailed ICD-10 catalog disease descriptions.

Supplementary material 2. a-f: Length-of-stay distribution by department. Vertical lines refer to median and threshold for prolonged length-of-stay computed by plos_threshold = median(X) + 1.48 * median (|X-median(X)|) = median(X) + MAD, where X is the train data of the respective department. For visual comparison of distributions, the axes were limited to y max. 1000 cases, x max. 200 days. Some outliers may not be displayed.


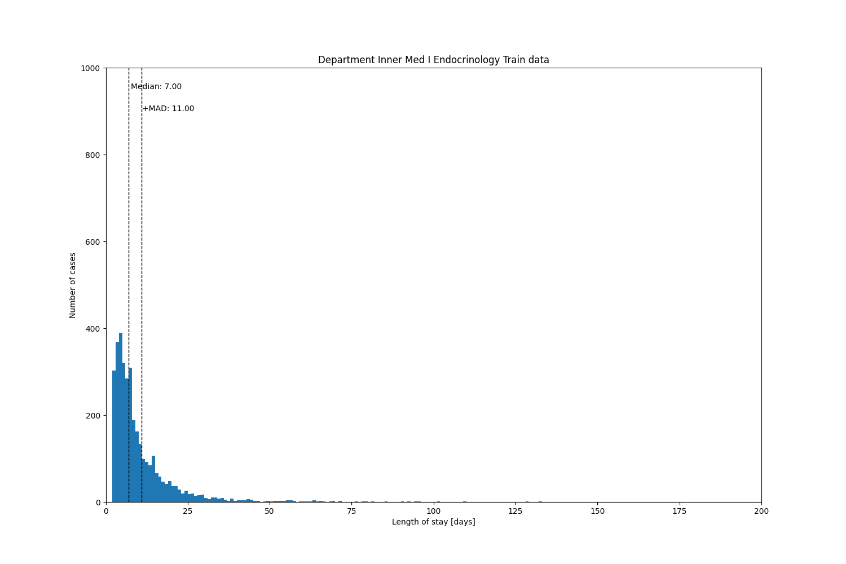
Supplementary material 2 a)


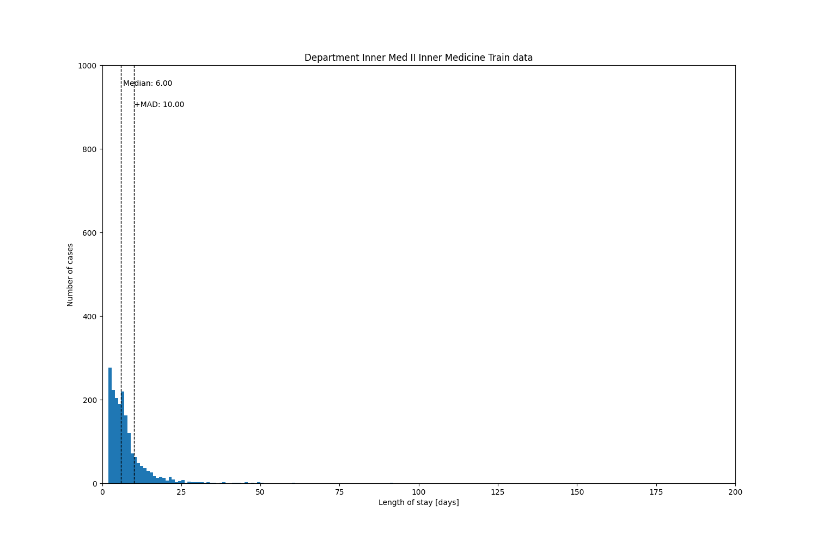
Supplementary material 2 b)


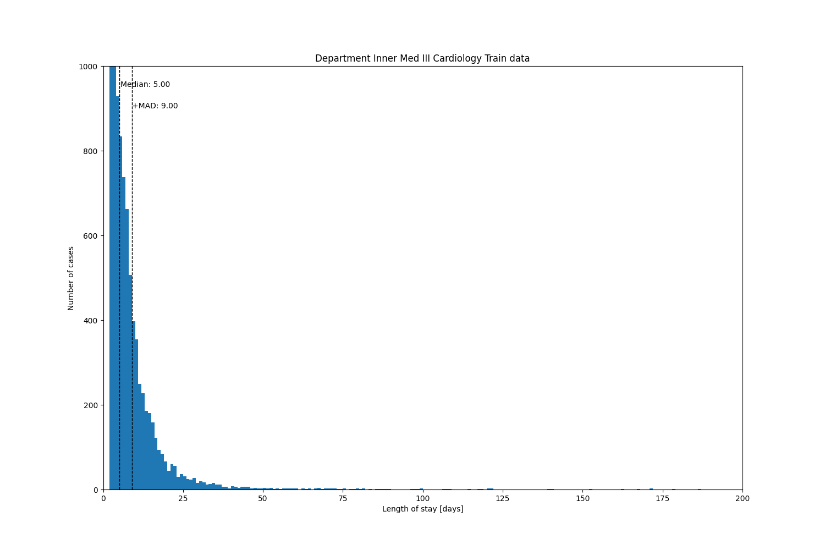
Supplementary material 2 c)


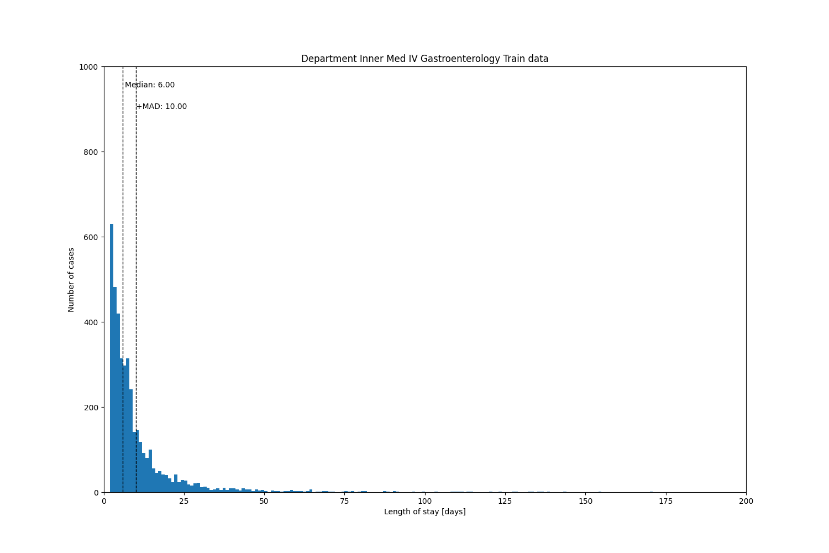
Supplementary material 2 d)

Supplementary material 2 e)


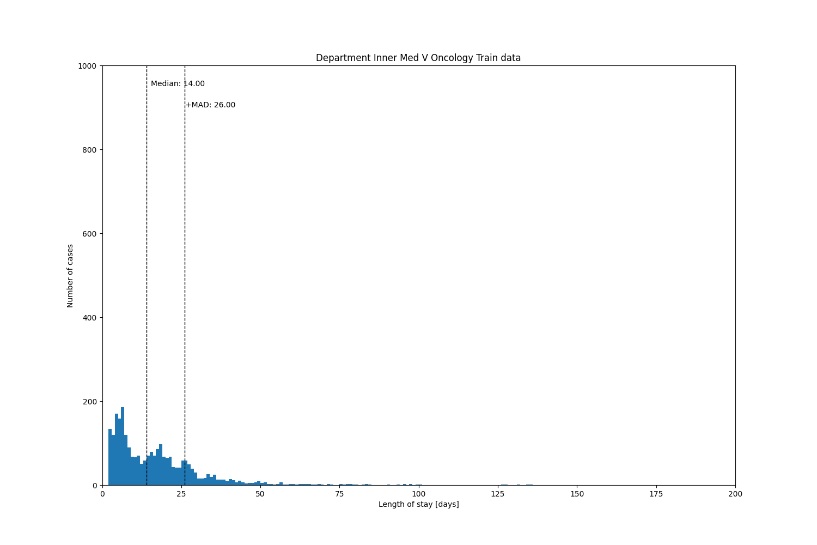


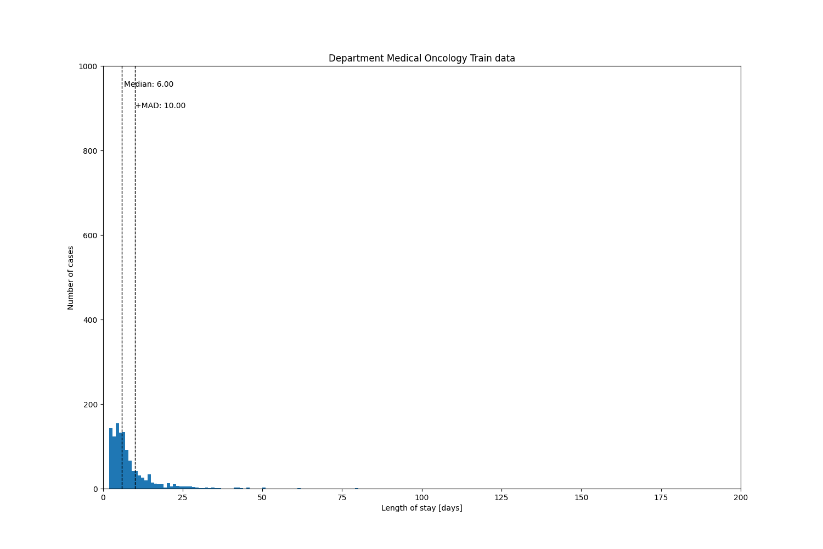
Supplementary material 2 f)

Supplementary material 3. Length-of-stay percentiles per department. Highlighted in bold are the corresponding values or ranges in which the MAD-based threshold lies. MAD: Median absolute deviation, s. formula in Supplementary material 2. Computed with midpoint interpolation as in the python command df_train.groupby("Diag_release_ou_spec")["los"].quantile(q=[0.5, 0.70, 0.75, 0.80, 0.85], interpolation='midpoint').

|  |  |  | length-of-stay percentile in train data | | | |
| --- | --- | --- | --- | --- | --- | --- |
| **Department** | **Pro-longed length-of-stay threshold MAD-based** | **50th median** | **70th** | **75th** | **80th** | **85th** |
| 1 Endocrinology | 11 | 7 | **10** | **12** | 14 | 16 |
| 2 General and Psychosomatics | 10 | 6 | 8 | 8 | **10** | 11 |
| 3 Cardiology | 9 | 5 | 8 | **9** | 11 | 13 |
| 4 Gastro-enterology | 10 | 6 | **10** | 11 | 13 | 16 |
| 5 Hematology | 26 | 14 | 20 | 23 | **25** | **28** |
| 6 Medical Oncology | 10 | 6 | 8 | **9** | **11** | 13 |


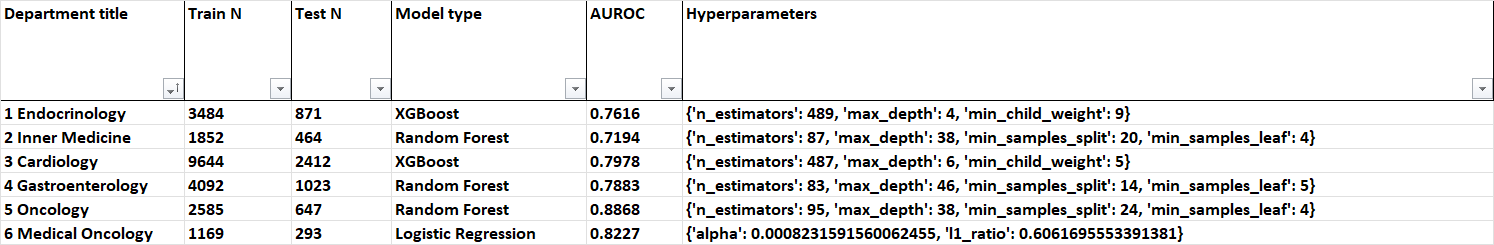
Supplementary material 4. Hyperparameters of best models by area under the receiver operator curve (AUROC) for prolonged length-of-stay.
