# Supplementary material for: Machine learning models incorporating somatic and mental comorbidities for prolonged length-of-stay prediction in a maximum care university hospital
Source: BMC Med Inform Decis Mak. 2025 Nov 26;25:436. doi: 10.1186/s12911-025-03290-3 (PMC12670805; doi:10.1186/s12911-025-03290-3)
Supplement: Supplementary file 5 — Supplementary Material 5 [file 12911_2025_3290_MOESM5_ESM.docx]

# Additional Files

## Additional File 1

File name: “Additional File 1.xlsx”

File format: .xlsx

Title: Descriptive statistics for departments.

Description: Table with descriptive statistics for departments by train and test set. Continuous variables reported as mean ± standard deviation [range]. Percentages refer to train or test set within departments, respectively. Demographics, main diagnosis, mental comorbidity and somatic comorbidity. LOS: length-of-stay.

## Additional File 2

File name: “Additional File 2.docx”

File format: .docx

Title: Supplementary Material.

Description:

Supplementary material 1. Details on derivation of WHO comorbidity groups.

Supplementary material 2. a-f: Length-of-stay distribution by department.

Supplementary material 3. Length-of-stay 70^th^-85^th^ percentiles by department.

Supplementary material 4. Hyperparameters of best models.

## Additional File 3

File name: “Additional File 3.xlsx”

File format: .xlsx

Title: Mean absolute SHAP values.

Description: Additional File 3. Ranks for mean absolute SHAP values for prediction of prolonged length-of-stay for models with best AUROC for all departments. Up to rank 30 displayed. Computed on train data. Color scaled by alphabetically sorted feature names so that each variable has the same color and its rank can be compared across departments.

## Additional File 4

File name: “Additional File 4.pdf”

File format: .pdf

Title: TRIPOD+AI checklist

Description: Additional File 4. TRIPOD + AI checklist filled for this study.
